# Progress in Diseases of the Nervous System

**Published:** 1901-06-22

**Authors:** 


### PROGRESS IN DISEASES OF THE NERVOUS SYSTEM.

**The Chemistry of Nerve Degeneration.**—Mott and Halliburton[1] found that in general-paralysis of the insane the marked degeneration that occurs in the brain is accompanied by the passing of the products of degeneration into the cerebro-spinal fluid. Of these nucleo-proteid and choline are most readily detected. Choline can also be found in the blood. They have subsequently found that these changes are not peculiar to general paralysis, but occur also in other degenerative nerve diseases, *e.g.*, disseminated sclerosis, alcoholic neuritis, beri-beri, and also in animals where the sciatic nerves had been divided and allowed to degenerate, just as in unilateral degeneration of the pyramidal tract, the degenerating nerves show a progressive increase in the per cent. of water and a diminution in phosphorus. The chemical explanation of the Marchi reaction is the replacement of phosphorised by non-phosphorised fat. When the Marchi reaction disappears in the later stages of degeneration the non-phosphorised fat has disappeared.

**Sensory Disturbances resulting from Cerebral Lesions.**—Verger[2] believes with Déjerine and Long that the sensory tract from the peduncle to the cortex is interrupted in the optic thalamus and thus is composed of two sets of neurons—bulbo-thalamic and thalamo-cortical. Hemianæsthesia may be due to a lesion interrupting either set of neurons. The thalamo-cortical fibres pass by the posterior limb of the internal capsule, and either this or the external nucleus of the thalamus is found to be the site of the lesion in cases of hemiplegia of central origin accompanied by sensory symptoms. Hemianæsthesia due to organic lesion is seldom so complete as the hysterical form; in particular the perception of pain persists in some degree even after extensive lesions. Verger believes, with Hermann Munk, that the cortical centres for sensibility are situated in the Rolandic region, and that in this region are received and registered tactile, muscular, and painful sensations, which constitute general sensibility and are necessary to the education of movement. He dissents entirely from Ferrier's localisation of perception in the hippocampal conditions.

**Surgical Treatment of Migraine.**—Whitehead[3] says that for the last twenty-five years he has never failed to treat successfully the most inveterate and severe cases of migraine by the introduction of a tape seton at the back of the neck. All the cases had been previously under medical treatment without receiving any material benefit. The last case he treated was that of a lady who had never for the last six years escaped many weeks without an attack of migraine of such severity that she had been prostrated with violent headache and sickness for periods extending from twelve to twenty-four hours. Since the introduction of a seton she had not had an attack for several months. The little operation may be done under nitrous-oxide anæsthesia. The skin of the back of the neck is grasped into a fold and a long-bladed scalpel is thrust through. A bodkin, threaded with a piece of household tape half an inch wide, is pushed through before the scalpel is withdrawn. Four inches of tape are left free at each end and tied together to prevent the tape being accidentally withdrawn. Instructions are given to the patient to move the tape from side to side each day. The seton ought to be worn uninterruptedly for three months at least in the first instance, and should the symptoms recur a second should be introduced.

[1] Brit. Med. Jour., Mar. 30, 1901. [2] Archiv. gén de Méd., Dec. 1900
[3] Brit. Med. Jour., Feb. 9, 1901.

